# Motivations for Social Distancing and App Use as Complementary Measures to Combat the COVID-19 Pandemic: Quantitative Survey Study

**DOI:** 10.2196/21613

**Published:** 2020-08-27

**Authors:** Kai Kaspar

**Affiliations:** 1 Department of Psychology University of Cologne Cologne Germany

**Keywords:** COVID-19, protection motivation theory, social distancing, contact tracing app, data donation app, social trust, data security

## Abstract

**Background:**

The current COVID-19 pandemic is showing negative effects on human health as well as on social and economic life. It is a critical and challenging task to revive public life while minimizing the risk of infection. Reducing interactions between people by social distancing is an effective and prevalent measure to reduce the risk of infection and spread of the virus within a community. Current developments in several countries show that this measure can be technologically accompanied by mobile apps; meanwhile, privacy concerns are being intensively discussed.

**Objective:**

The aim of this study was to examine central cognitive variables that may constitute people’s motivations for social distancing, using an app, and providing health-related data requested by two apps that differ in their direct utility for the individual user. The results may increase our understanding of people’s concerns and convictions, which can then be specifically addressed by public-oriented communication strategies and appropriate political decisions.

**Methods:**

This study refers to the protection motivation theory, which is adaptable to both health-related and technology-related motivations. The concept of social trust was added. The quantitative survey included answers from 406 German-speaking participants who provided assessments of data security issues, trust components, and the processes of threat and coping appraisal related to the prevention of SARS-CoV-2 infection by social distancing. With respect to apps, one central focus was on the difference between a contact tracing app and a data donation app.

**Results:**

Multiple regression analyses showed that the present model could explain 55% of the interindividual variance in the participants’ motivation for social distancing, 46% for using a contact tracing app, 42% for providing their own infection status to a contact tracing app, and 34% for using a data donation app. Several cognitive components of threat and coping appraisal were related to motivation measurements. Trust in other people’s social distancing behavior and general trust in official app providers also played important roles; however, the participants’ age and gender did not. Motivations for using and accepting a contact tracing app were higher than those for using and accepting a data donation app.

**Conclusions:**

This study revealed some important cognitive factors that constitute people’s motivation for social distancing and using apps to combat the COVID-19 pandemic. Concrete implications for future research, public-oriented communication strategies, and appropriate political decisions were identified and are discussed.

## Introduction

### Background

The World Health Organization has declared the outbreak of COVID-19 to be a global pandemic [1]. Development of therapeutics and vaccines started early and remains a high priority [2,3]; however, no effective vaccine or drug treatment is currently available [4]. Moreover, negative social and economic consequences of broader shutdowns in many countries are already visible [5]; therefore, measures are being taken to revive social and economic life. The most critical and challenging task is to revive public life while minimizing the risk of infection. In addition to hand hygiene and mask-wearing [6], reducing interactions between people by social distancing is an effective and prevalent public health measure to reduce the risk of infection and spread of the virus within a community [7]. Current developments in several countries show that this measure may be technologically accompanied by mobile apps. Indeed, app stores already offer a wide range of apps related to the current pandemic; meanwhile, privacy concerns are being intensively discussed [8]. Hence, current research is focusing on ethical aspects of contact tracing apps [9,10] and ethical guidelines for such apps have already been formulated [11]. At the same time, an increasing number of national governments are disseminating contact tracing apps to help contain the pandemic; these apps differ remarkably regarding their technological approaches [12]. Importantly, the utility of mobile apps has already been examined in the context of previous epidemics, such as the Ebola epidemic in West Africa between 2014 and 2016 [13,14]. Given the current relevance of social distancing and using mobile apps as a complementary measure to combat the COVID-19 pandemic, in this study, I aimed to examine central cognitive variables that may constitute people’s motivation for social distancing, using an app, and providing health-related data requested by two apps that differ in their direct utility for the individual user. The results would help increase our understanding of people’s concerns and convictions, which can then be specifically addressed by public-oriented communication strategies and appropriate political decisions.

One of the most prominent technological concepts during the pandemic is contact tracing, such as the app concept developed by the Pan-European Privacy-Preserving Proximity Tracing (PEPP-PT) initiative. In general, the Bluetooth connection of a smartphone is used “in order to detect whether two people have come into close enough physical proximity to risk an infection [15].” The app notifies users when they have had critical contact (in terms of time span and spatial proximity) with a person infected with SARS-CoV-2, the virus that causes COVID-19, so that the users can take appropriate measures. Importantly, each individual app user voluntarily provides their infection status and should not be identifiable by other app users because a critical contact is signaled with a temporal delay.

A second app type is offered by the Robert Koch Institute, which is the central institution of the German Federal Government in the field of disease surveillance and prevention. The app is officially called the Corona Data Donation app (Data Donation app) and it is connected to the individual user’s digital wearables (eg, smartwatches and fitness trackers); it continuously tracks data related to the user’s health and daily activities (eg, heart rate and sleep rhythm) and additional personal data, including postal code, weight, age, and gender. According to the official app description, identification of app users is not possible. However, the individual user receives no direct benefit from using this app type, as it does not provide any feedback. Instead, all data are collected centrally by the Robert Koch Institute to create different maps that may indicate and facilitate specific local measures. Importantly, using the Data Donation app and providing the personal data requested by this app are basically the same. In contrast, using a contact tracing app and voluntarily providing one’s own infection status to that app are independent measures. It appears to be important to focus on these very different functional accounts to examine whether users’ evaluation and use motivation are specific or general.

### The Protection Motivation Theory and Social Distancing

The present study refers to the protection motivation theory (PMT). This theory was originally developed on the basis of expectancy-value approaches in the context of health sciences to explain preventive behavior based on threat and coping appraisal processes [16]. Meta-analyses [17,18] showed that the components of the theory reliably explain protection motivation. More recent studies supported these findings in several contexts that are not limited to health-related issues, such as skin cancer prevention [19] or people’s intention to receive a seasonal influenza vaccination [20]. For example, Tsai et al [21] successfully applied the PMT to internet users’ motivation for enacting safety precautions, Marett et al [22] used it to explain adaptive and maladaptive responses to risks associated with posting personal information on social networking sites, and Vance et al [23] found that most components of the PMT were significantly related to employees’ intention to comply with information security policies. Moreover, a meta-analysis [24] revealed that the PMT is particularly effective in the context of information security behavior if the behavior is voluntary and specific and the potential security threat is directed to the individual instead of other people or the person’s organization. These conditions are met with respect to the use of a contact tracing app and the Data Donation app. Thus, the PMT is a very powerful and flexible theory that is adaptable to both health-related and technology-related motivations, which qualifies it for the present study.

In general, the PMT comprises threat appraisal of the potential risk (eg, infection with SARS-CoV-2) and coping appraisal of the recommended preventive behavior (eg, social distancing). Threat appraisal includes the perceived severity of and vulnerability to the negative consequences of maladaptive behavior. The more pronounced these two variables, the higher the motivation to perform the recommended behavior. Threat appraisal also includes the perceived rewards associated with not performing the recommended behavior, counteracting protection motivation. Coping appraisal includes the perceived self-efficacy and response efficacy of the recommended behavior, which positively affect an individual’s intention to actively prevent risks. Coping appraisal also includes the perceived response costs, which counteract protection motivation. With respect to the prevention of SARS-CoV-2 infection by social distancing, the following hypotheses resulted:

H1a: The perceived severity of an infection is positively related to the motivation for social distancing.

H1b: The perceived vulnerability to an infection is positively related to the motivation for social distancing.

H1c: The perceived rewards associated with avoiding social distancing are negatively related to the motivation for social distancing.

H2a: The self-efficacy regarding social distancing is positively related to the motivation for social distancing.

H2b: The perceived response efficacy of social distancing is positively related to the motivation for social distancing.

H2c: The perceived response costs of social distancing are negatively related to the motivation for social distancing.

**Figure 1:**
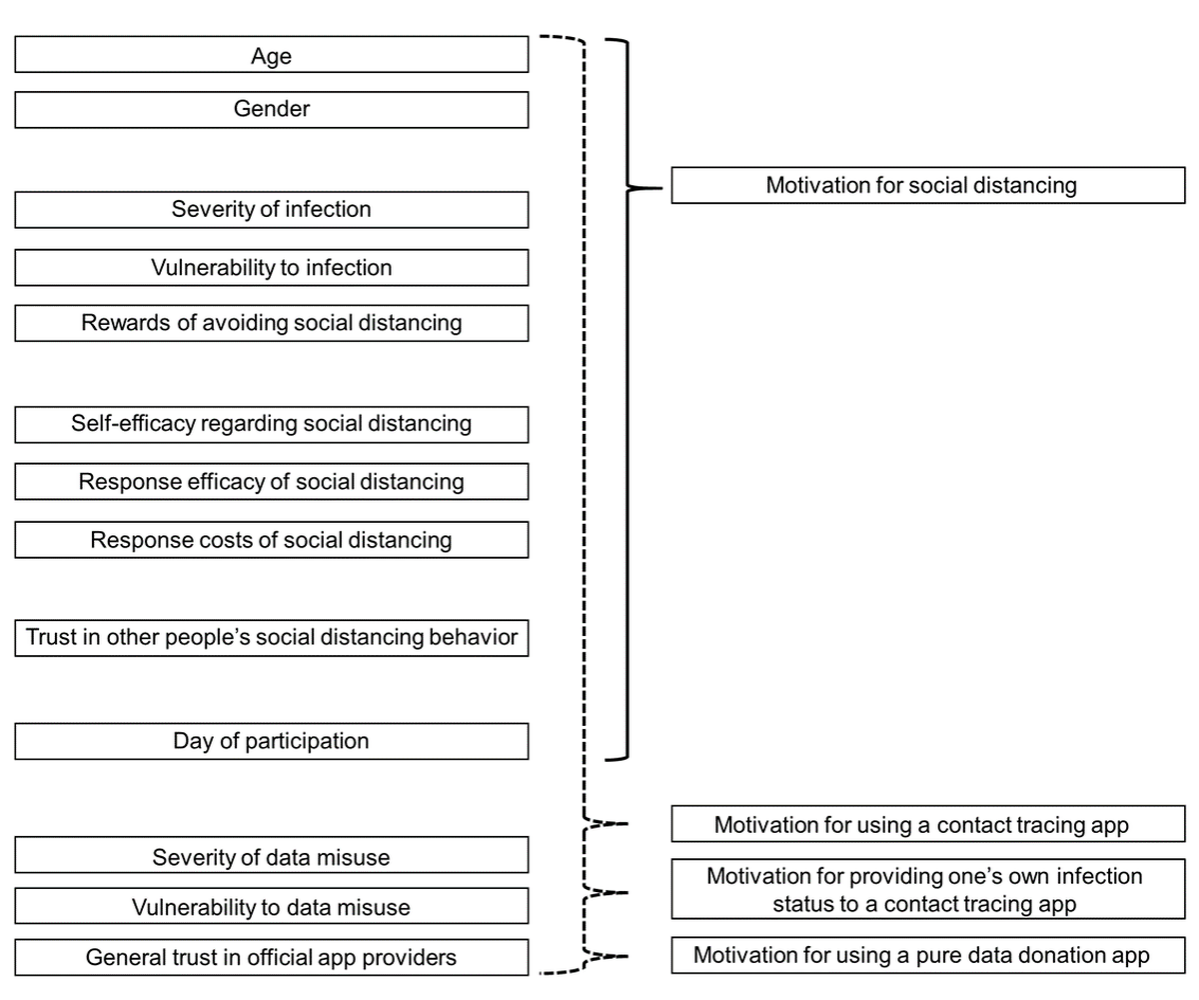
The regression models examined in the present study, with independent variables on the left side and dependent variables on the right side.

In addition to these standard PMT variables, trust was added (see Figure 1). Rousseau et al [25] defined trust as “a psychological state comprising the intention to accept vulnerability based upon positive expectations of the intentions or behavior of another.” Indeed, because social distancing is a measure that only works effectively when performed collectively, trust in other people’s social distancing behavior appears to be a critical component. However, two phenomena are conceivable: in terms of the social exchange theory [26] and the concept of reciprocity [27], higher trust in others’ willingness to adequately perform social distancing may increase one’s own motivation for social distancing. This relation would reflect the benefits of solidarity required to combat the current pandemic [28,29]. Alternatively, and in terms of a compensation mechanism to reduce one’s own infection risk, a negative relationship between trust in others’ social distancing behavior and one’s own protection motivation is conceivable. Thus, the following undirected hypothesis was formulated:

H3a: Trust in other people’s social distancing behavior is related to one’s own motivation for social distancing.

### Motivations for Using an App and Providing Personal Data

Regarding the use of an app and the provision of personal data, the question arises of whether the corresponding motivations are related to the cognitive variables assumed to constitute protection motivation or whether these are completely different evaluation processes. Depending on the answer to this question, an appropriate public communication strategy can be developed that supports realistic assessments and acceptance of different app types. Given that the main purpose of a contact tracing app is informing the user of critical contacts with infected persons, the perceived severity of and vulnerability to SARS-CoV-2 infection may be positively related to the motivation for using such an app. Perceived rewards of avoiding social distancing may also be associated with app use motivation. In contrast, coping appraisal of social distancing should not be related to the motivation for using a contact tracing app. In general, neither a contact tracing app nor a data donation app can actually help individual users to actively prevent infection. With respect to the motivation for providing personal data to both app types, threat and coping appraisal should not show a relationship because providing this information is only useful for other users (contact tracing app) or researchers and policy makers (data donation app). Thus, the following open research questions were formulated:

RQ1a: Are the motivations for app use and data provision related to the threat appraisal of SARS-CoV-2 infection?

RQ1b: Are the motivations for app use and data provision related to the coping appraisal of social distancing?

Moreover, and with respect to the idea of a compensation mechanism outlined above, reduced trust in other people’s social distancing behavior may also be associated with increased motivation to use a contact tracing app, as this type of app can help the user monitor their own risk of being infected.

H3b: Trust in other people’s social distancing behavior is negatively related to one’s motivation for using a contact tracing app.

Additionally, the present PMT model was extended by threat and trust variables that are specifically tailored to app use (see Figure 1). In line with previous studies applying the PMT to data security issues [30,31], severity of data misuse and vulnerability to data misuse were added when focusing on the motivation for app use and the provision of personal data. Woon et al [31] found that perceived severity but not vulnerability was related to wireless network security measures. Banks et al [30] found that perceived threat was negatively associated with the intention to share personal information on web-based social media platforms, while perceived severity and vulnerability were both positively related to threat appraisal. Accordingly, the following hypotheses were tested:

H4a: The perceived severity of data misuse is negatively related to the motivations for using a contact tracing app and for using the Data Donation app.

H4b: The perceived vulnerability to data misuse is negatively related to the motivations for using a contact tracing app and for using the Data Donation app.

H4c: The perceived severity of data misuse is negatively related to the motivation for voluntarily providing one’s own infection status to a contact tracing app.

H4d: The perceived vulnerability to data misuse is negatively related to the motivation for voluntarily providing one’s own infection status to a contact tracing app.

Applying the trust construct to app use addresses the users’ trust in the providers of an app that collects personally relevant information. Indeed, Lo et al [32] found that trust in social networking sites was positively related to users’ willingness to provide personal information, leading to the following hypotheses:

H5a: General trust in official app providers with respect to the use, management, and protection of user data is positively related to the motivation for using both a contact tracing app and the Data Donation app.

H5b: General trust in official app providers with respect to the use, management, and protection of user data is positively related to the motivation for voluntarily providing one’s own infection status to a contact tracing app.

The present study focuses on two fundamentally different app types. Although neither app type helps to reduce the user’s individual risk of infection, a contact tracing app obviously has some personal utility, whereas the Data Donation app has no direct utility for its users. Consequently, motivation and acceptance should be higher for the more personally useful contact tracing app:

H6a: Motivation for use is higher for a contact tracing app compared to the Data Donation app.

H6b: Motivation for providing personal data is higher for a contact tracing app (infection status) compared to the Data Donation app (health, activity, and personal data).

H6c: The acceptance of mandatory use would be higher for a contact tracing app compared to the Data Donation app.

Finally, as shown in Figure 1, age was included in the regression models examined here due to older people’s higher risk of a severe course of disease elicited by the novel coronavirus [33] and the ongoing public discussion about this risk [34]. Gender was included due to the well-known gender differences in health-related behavior [35]. Finally, the date of participation in this study was considered, as the pandemic was in progress; consequently, subjective risk assessment may change and habituation effects may occur over time.

## Methods

### Participants

The study included a final data set of 406 German-speaking participants (290 women, 71.4%) with a mean age of 32.56 years (SD 13.76). I previously excluded 9 participants: 3 (33%) were excluded due to incomplete data, 2 (22%) were younger than the required minimum age of 18 years for participation, and 4 (44%) reported their gender as “diverse,” which was an insufficient subsample for the gender-related statistical analyses. The highest educational attainment that was reported most often by the 406 participants was a higher education entrance qualification (173, 42.6%), followed by a master’s degree or diploma (93, 22.9%), a bachelor’s degree (70, 17.2%), completed vocational training (41, 10.1%), a secondary school certificate (21, 5.2%), no complete school leaving certificate (6, 1.5%), and main school graduation (2, 0.5%). At the time of the study, 385 of the 406 participants (94.8%) were not using any apps related to COVID-19, while 21 participants (5.2%) had already used the Data Donation app provided by the Robert Koch Institute. Importantly, a contact tracing app did not yet exist but had been officially announced for Germany at the time of the study. The participants were recruited through convenience sampling. The link to the study was broadly disseminated via mailing lists, social media, and a survey platform of a national journal (*Psychologie Heute*). Participation in the study was voluntary, and no incentives were provided. No identifying data were collected to guarantee the anonymity of the participants. At the start of the study, the participants were informed about the purpose of the study, that all data would be processed only for research purposes, that they would remain anonymous, and that they could prematurely stop the study at any point in time. In the latter case, the participant’s data were deleted from the final data set before the analyses were performed. The participants finally indicated informed consent by clicking a corresponding box. In Germany, as stated by the German Research Association (Deutsche Forschungsgemeinschaft, DFG), ethics committee approval was not required for this survey because the research did not include a treatment, did not pose any threats or risks to the respondents, and was not associated with high physical or emotional stress; also, the respondents were informed about the objectives of the survey. The study ran for 30 days, starting on April 15, 2020.

### Procedure

Participants initially provided their gender, age, and highest educational qualification. Afterward, the concept and functions of the two app types were presented in a detailed summary according to official descriptions of the PEPP-PT contact tracing app and the Data Donation app, as outlined above. With respect to each of the two apps, participants answered some questions related to app use. They subsequently assessed data security issues in terms of the perceived severity of potential misuse of their data, their perceived vulnerability to data misuse, and their general trust in official app providers with respect to the use, management, and protection of user data. Then, the participants reported their protection motivation by social distancing and their trust in other people’s social distancing behavior. Finally, they assessed all PMT variables covered by threat appraisal (severity, vulnerability, rewards) and coping appraisal (self-efficacy, response efficacy, response costs) related to the prevention of SARS-CoV-2 infection by social distancing.

### Measures

#### Questions Related to the Apps

Based on a 7-point scale (1=“not motivated at all” to 7=“very motivated”), participants indicated how much they were motivated to voluntarily use the described app and to voluntarily provide the personal data requested by the app. They also responded to the question “How much would you like it if the use of this app became a mandatory requirement for everyone?” (1=“not at all” to 7=“very”) and whether they were already using the existing Data Donation app (yes/no). All subsequent measures were based on three items each with 7-point rating scales (1=“completely disagree” to 7=“completely agree”). All rating scales were continuously numbered from 1 to 7 and had verbal markers at the endpoints.

#### Data Security Issues

Items adapted from Banks et al [30] and Dang-Pham and Pittayachawan [36] were used to assess the participants’ perceived severity of potential misuse of their data (eg, “If my personal information collected by a coronavirus app would be misused, it could harm me,” Cronbach α=.80) and the perceived vulnerability to data misuse (eg, “I feel that I am vulnerable to misuse of my personal information collected by a coronavirus app,” α=.76). Items adapted from Lo [32] were used to assess the participants’ general trust in official providers of a COVID-19 app with respect to the use, management, and protection of user data (eg, “I believe that official providers of coronavirus apps are genuine and sincere in managing my personal information,” α=.93).

#### Motivation for Social Distancing and Trust in Others’ Social Distancing Behavior

Items adapted from Kaspar [37] assessed participants’ protection motivation for social distancing (eg, “Over the next few weeks, I will avoid physical proximity to people who do not live in my household,” α=.80). Trust in other people’s intention to adequately perform social distancing behavior was measured by items adapted from Ross et al [38] (eg, “I think most people are currently trying their best to avoid getting too close to other people in public life so that the coronavirus cannot spread further,” α=.80).

#### Threat and Coping Appraisal Regarding COVID-19

Items from previous studies [23,36,37,39,40] were adapted to social distancing behavior. Measures of threat appraisal included the perceived severity of an infection (eg, “If I became infected with the coronavirus, it would have a strong negative effect on my health,” α=.89), the perceived vulnerability to an infection if no social distancing was performed (eg, “Other infected people will infect me with the coronavirus if I do not keep appropriate physical distance from them,” α=.74), and perceived intrinsic rewards associated with not performing social distancing from people who do not live in the participant’s household (eg, “I currently enjoy meeting with other people who do not live in my household,” α=.91). Coping appraisal included the participants’ perceived self-efficacy regarding social distancing (eg, “At the moment, it is easy for me to create physical distance to people who do not live in my household,” α=.62), response efficacy in terms of the effectiveness of social distancing in averting an infection (eg, “Keeping sufficient distance from other people in public life protects me from the coronavirus,” α=.82), and perceived response costs (eg, “At the moment, I find it exhausting to create sufficient spatial distances to other people in public space,” α=.66).

## Results

### Intercorrelations and Mean Values of Independent Variables

In sum, intercorrelations among the independent variables of the regression models were rather low, with few exceptions (Table 1). Age and gender showed almost no significant correlation with the PMT or trust variables. The highest correlation was between age and the perceived severity of infection. Perceived response efficacy of social distancing showed several high correlations with other PMT variables. In accordance with the theoretical structure of threat and coping appraisal, perceived rewards and response costs were positively correlated with each other; however, they showed negative correlations with all other PMT variables (severity, vulnerability, self-efficacy, and response efficacy). Trust in other people’s social distancing behavior was weakly positively correlated with self-efficacy and response efficacy. Perceived severity of and vulnerability to data misuse were highly positively correlated with each other but negatively correlated with general trust in app providers. Perceived vulnerability to infection and response efficacy of social distancing were positively correlated with general trust in app providers. Interestingly, the day on which the respondents participated in the survey, reflecting the temporal progress of the pandemic, was positively correlated with perceived rewards associated with avoiding social distancing but negatively correlated with self-efficacy regarding social distancing and trust in other people’s social distancing behavior.

One-sample *t* tests showed that the mean value of most of the independent variables was above the midpoint of the 7-point scales (Table 2), except for the perceived severity of an infection (no deviation from the midpoint of the scale) and perceived rewards of avoiding social distancing (below the midpoint of the scale). The self-efficacy and response efficacy of social distancing were rated particularly high.

**Table 1:** Bivariate correlations (Pearson *r* and two-tailed *P* value) among all independent variables of the regression models.

Variable		1.	2.	3.	4.	5.	6.	7.	8.	9.	10.	11.	12.	13.
**1. Age**														
	*r*	1	–.02	.29	.08	–.13	.23	.06	.04	.12	–.08	.09	.10	–.05
	*P* value	—^a^	.66	<.001	.11	.01	<.001	.20	.45	.01	.13	.08	.04	.37
**2. Gender^b^**														
	*r*	–.02	1	.04	.06	–.11	.06	.06	.07	.05	.02	.01	–.05	.01
	*P* value	.66	—	.41	.23	.03	.24	.21	.19	.37	.66	.83	.28	.92
**3. Severity of infection**														
	*r*	.29	.04	1	.44	–.17	.19	.25	–.14	–.07	.04	.17	.05	.11
	*P* value	<.001	.41	—	<.001	.001	<.001	<.001	.004	.15	.39	.001	.32	.03
**4. Vulnerability to infection**														
	*r*	.08	.06	.44	1	–.31	.25	.52	–.12	.05	.03	.01	–.05	.25
	*P* value	.11	.23	<.001	—	<.001	<.001	<.001	.01	.32	.53	.79	.36	<.001
**5. Rewards of avoiding social distancing**														
	*r*	–.13	–.11	–.17	–.31	1	–.37	–.43	.28	–.08	.17	.10	.12	–.14
	*P* value	.01	.03	.001	<.001	—	<.001	<.001	<.001	.11	.001	.045	.02	.006
**6. Self-efficacy regarding social distancing**														
	*r*	.23	.06	.19	.25	–.37	1	.50	–.25	.16	–.17	.05	–.03	.10
	*P* value	<.001	.24	<.001	<.001	<.001	—	<.001	<.001	.002	<.001	.30	.58	.05
**7. Response efficacy of social distancing**														
	*r*	.06	.06	.25	.52	–.43	.50	1	–.21	.15	–.04	–.06	–.16	.31
	*P* value	.20	.21	<.001	<.001	<.001	<.001	—	<.001	.003	.43	.23	.001	<.001
**8. Response costs of social distancing**														
	*r*	.04	.07	–.14	–.12	.28	–.25	–.21	1	–.07	.06	.12	.07	–.07
	*P* value	.45	.19	.004	.01	<.001	<.001	<.001	—	.18	.20	.01	.15	.15
**9. Trust in other people’s social distancing behavior**														
	*r*	.12	.05	–.07	.05	–.08	.16	.15	–.07	1	–.17	.02	–.01	.10
	*P* value	.01	.37	.15	.32	.11	.002	.003	.18	—	.001	.74	.87	.052
**10. Day of participation**														
	*r*	–.08	.02	.04	.03	.17	–.17	–.04	.06	–.17	1	.05	.05	–.001
	*P* value	.13	.66	.39	.53	.001	<.001	.43	.20	.001	—	.28	.33	.98
**11. Severity of data misuse**														
	*r*	.09	.01	.17	.01	.10	.05	–.06	.12	.02	.05	1	.53	–.25
	*P* value	.08	.83	.001	.79	.045	.30	.23	.01	.74	.28	—	<.001	<.001
**12. Vulnerability to data misuse**														
	*r*	.10	–.05	.05	–.05	.12	–.03	–.16	.07	–.01	.05	.53	1	–.55
	*P* value	.04	.28	.32	.36	.02	.58	.001	.15	.87	.33	<.001	—	<.001
**13. General trust in official app providers**														
	*r*	–.05	.01	.11	.25	–.14	.10	.31	-.07	.10	–.001	–.25	–.55	1
	*P* value	.37	.92	.03	<.001	.006	.05	<.001	.15	.052	.98	<.001	<.001	—

^a^—: not applicable.

^b^0=male, 1=female.

**Table 2:** Descriptive statistics and results of one-sample t tests against the scales’ midpoint value (4) for independent variables of the regression models (age, gender, and day of participation were excluded due to the inappropriateness of the statistics in these cases).

Variable	Mean (SD)	*t*_405_	*P* value	Cohen *d*
Severity of infection	4.01 (1.55)	0.139	.89	0.01
Vulnerability to infection	4.85 (1.46)	11.679	<.001	0.58
Rewards of avoiding social distancing	2.57 (1.60)	–17.976	<.001	0.89
Self-efficacy regarding social distancing	6.02 (1.07)	38.148	<.001	1.89
Response efficacy of social distancing	5.92 (1.17)	33.194	<.001	1.64
Response costs of social distancing	4.48 (1.48)	6.526	<.001	0.32
Trust in other people’s social distancing behavior	5.10 (1.21)	18.372	<.001	0.91
Severity of data misuse	5.09 (1.52)	14.475	<.001	0.72
Vulnerability to data misuse	4.96 (1.43)	13.442	<.001	0.67
General trust in official app providers	4.20 (1.65)	2.410	.02	0.12

### Motivation for Social Distancing

In the next step, multiple regression analyses were conducted (Table 3). Initially, the assumptions of the linear regression model [41] were checked and met for all models, except for one case of pronounced heteroscedasticity (see Multimedia Appendix 1). Hence, significance testing was based on the heteroscedasticity-robust HC3 estimator in this case [42], while the standard ordinary least squares (OLS) estimator was preferred in cases of homoscedasticity [43]. The participants’ motivation for social distancing, serving as a dependent variable, showed a positive relation to the perceived severity of an infection (supporting H1a), no relation to the perceived vulnerability to an infection (contradicting H1b), and a negative relation to perceived rewards associated with avoiding social distancing (supporting H1c). Self-efficacy and response efficacy of social distancing were positively and strongly related to the motivation for social distancing (supporting H2a and H2b), whereas perceived response costs were unrelated (contradicting H2c). Finally, trust in other people’s social distancing behavior was positively related to participants’ motivation for social distancing (supporting H3a), whereas the day of participation, age, and gender were nonrelated. Overall, the model explained 55% of the interindividual variance in participants’ motivation for social distancing. Importantly, all independent variables except gender showed significant bivariate correlations with the motivation for social distancing; however, several of these significant relationships disappeared in the complete regression model that simultaneously considered all independent variables (for bivariate correlations, see Multimedia Appendix 1).

**Table 3:** Results of the multiple regression analyses with standardized coefficients (β) and *P* values based on the heteroscedasticity-robust HC3 estimator (P_HC3_) or standard OLS estimates (P_OLSE_).

Independent variable	Motivation for social distancing (*R*^2^=.547, *P*<.001)		Motivation for using a contact tracing app (*R*^2^=.457, *P*<.001)		Motivation for providing the infection status to a contact tracing app (*R*^2^=.423, *P*<.001)		Motivation for using the Data Donation app (*R*^2^=.344, *P*<.001)	
	β	*P_HC3_* ^a^	β	*P_OLSE_* ^b^	β	*P_OLSE_*	β	*P_OLSE_*
Age	–.034	.34	–.021	.61	–.014	.74	–.088	.05
Gender^c^	–.015	.70	–.047	.22	–.007	.85	.027	.52
Severity of infection	.117	.003	.077	.09	.027	.56	.039	.43
Vulnerability to infection	–.014	.74	.072	.14	.042	.40	.015	.77
Rewards of avoiding social distancing	–.254	<.001	–.017	.70	–.051	.26	–.058	.23
Self-efficacy regarding social distancing	.211	<.001	.128	.006	.089	.06	.008	.88
Response efficacy of social distancing	.401	<.001	.103	.045	.098	.07	.092	.11
Response costs of social distancing	.029	.41	.137	.001	.056	.18	.040	.37
Trust in other people’s social distancing behavior	.118	.003	–.078	.046	–.087	.03	–.103	.02
Day of participation	–.025	.53	–.042	.29	–.012	.77	–.027	.53
Severity of data misuse	N/A^d^	N/A	–.098	.03	–.075	.11	–.070	.16
Vulnerability to data misuse	N/A	N/A	–.219	<.001	–.224	<.001	–.195	.001
General trust in official app providers	N/A	N/A	.379	<.001	.384	<.001	.353	<.001

^a^*P_HC3_*: *P* value based on the heteroscedasticity-robust HC3 estimator.

^b^*P_OLSE_*: *P* value based on the standard ordinary least squares estimate.

^c^0=male, 1=female.

^d^Not applicable.

### Motivations for Using a Contact Tracing App and the Data Donation App

Regarding participants’ motivation for using an app (Table 3), the multiple regression analyses revealed some differences between the contact tracing app and the Data Donation app. Independently of the app type, there was no relation between use motivation and the components of threat appraisal of SARS-CoV-2 infection (severity, vulnerability, and rewards) (RQ1a). In contrast, all components of coping appraisal (self-efficacy, response efficacy, and response costs) were positively related to the motivation for using a contact tracing app but not related to the motivation for using the Data Donation app (RQ1b). Trust in other people’s social distancing behavior was negatively related to the motivation for using a contact tracing app (supporting H3b) but also to the motivation for using the Data Donation app (not predicted). Perceived severity of data misuse was negatively and exclusively related to the motivation for using the contact tracing app (partially supporting H4a), while age was negatively and exclusively related to the motivation for using the Data Donation app (not predicted). Perceived vulnerability to data misuse was negatively and strongly related to the motivations for using both app types (supporting H4b). Also, general trust in official app providers showed a positive and the most pronounced relation to the motivations for using both app types (supporting H5a). The participants’ gender and the day of participation in the study were not related to app use motivation. The models explained 46% and 34% of the interindividual variance in the participants’ motivation for using a contact tracing app and the Data Donation app, respectively.

### Motivation for Providing One’s Own Infection Status to a Contact Tracing App

In the final regression analysis, the participants’ motivation for voluntarily providing their own infection status to a contact tracing app served as the dependent variable (Table 3). Threat appraisal of an infection (RQ1a) and coping appraisal of social distancing (RQ1b) were not significantly related to the motivation for providing one’s own infection status, as expected. Trust in other people’s social distancing behavior was negatively related to the motivation for providing the infection status to the contact tracing app (not predicted). Perceived severity of data misuse did not show a significant relation to participants’ willingness to share their infection status (contradicting H4c); however, perceived vulnerability to data misuse showed a negative relationship (supporting H4d). Moreover, general trust in official app providers showed a positive and strong relationship to the motivation for providing the infection status (supporting H5b). Again, the participants’ gender and the day of participation in the study were not related to the motivational dependent variable. The explained variance was 42%.

### Use Motivation and Acceptance of App Types

Finally, *t* tests for paired samples revealed that the motivation for using an app was higher for the contact tracing app (mean 4.11, SD 2.24) than for the Data Donation app (mean 3.76, SD 2.13; t_405_=3.72, *P*<.001, Cohen *d*=0.18) (supporting H6a). The motivation for providing the personal data requested by the individual app type was also higher in the case of the contact tracing app (mean 4.48, SD 2.32) compared to the Data Donation app (mean 3.41, SD 2.23; t_405_=10.86, *P*<.001, *d*=0.54) (supporting H6b). Also, participants were more receptive to the idea of the contact tracing app becoming mandatory (mean 3.06, SD 2.17) compared to the Data Donation app (mean 2.65, SD 1.98; t_405_=6.57, *P*<.001, *d*=0.33) (supporting H6c).

## Discussion

The results of the present study show that the PMT is a useful model to explain people’s motivation to protect themselves from SARS-CoV-2 infection by social distancing and to use apps related to the COVID-19 pandemic. Although previous research [44] showed that a motivation-behavior gap can occur because “people do not always do the things that they intend to do,” motivation supports effective health measures.

### Social Distancing

Threat-appraisal of potential infection was related to the motivation for social distancing. Perceived severity of infection was positively related to the motivation for social distancing; however, perceived vulnerability was not. This result indicates that the perceived severity of an infection with SARS-CoV-2 is more important for social distancing motivation than the perceived vulnerability when both variables are simultaneously considered. Also, perceived intrinsic rewards of not performing social distancing showed a strong negative relationship to protection motivation, indicating that perceived social benefits from being physically surrounded by others can counteract prevention strategies. Hence, it appears to be important to develop alternative and appropriate means to satisfy people’s social needs during the pandemic. Indeed, the longer the pandemic and the restrictions associated with it last, the more the commitment of the population to prevention strategies may decrease. The day of participation in this study, reflecting the temporal progress of the pandemic, was positively correlated with perceived rewards associated with not social distancing but negatively correlated with self-efficacy regarding social distancing and trust in other people’s social distancing behavior. A more encouraging result is that coping appraisal of social distancing, namely self-efficacy and response efficacy, showed strong positive relationships to the motivation for social distancing and were rated above average. Hence, it appears it would be fruitful to foster these factors with health campaigns. At the same time, perceived costs of social distancing were not related to protection motivation but were also rated above average. In contrast, the perceived rewards of avoiding social distancing were rated below average; this draws a somewhat contradictory picture that should be scrutinized in further research. Finally, and in line with social exchange theory [26] and the concept of reciprocity [27], participants’ trust in other people’s social distancing behavior was positively related to their own motivation for social distancing. This result is promising, as it supports the relevance of solidarity required to combat the current pandemic [28,29].

### App Use

It is important to note two aspects once more. First, while using the Data Donation app and providing the personal data requested by this app are basically the same, using a contact tracing app and voluntarily providing one’s own infection status to this app are independent measures. Second, although neither app type helps reduce the user’s individual risk of infection, a contact tracing app has some personal utility because it can help an individual monitor their own risk of being infected, whereas the Data Donation app has no direct utility for its users. Given these conceptual and technical differences, threat-appraisal of potential infection was not related to the motivation for using either app type or for providing one’s own infection status to a contact tracing app. However, all components of coping appraisal of social distancing were positively related to the motivation for using a contact tracing app but were not related to using the Data Donation app. Self-efficacy and the response efficacy of social distancing were positively correlated to the motivation for using a contact tracing app, indicating that people who believe in their coping skills and the effectiveness of the recommended coping strategy tend to support further measures to combat the pandemic. At the same time, the participants appeared to be interested in effective ways to combat the pandemic overall, as the motivation for using a contact tracing app increased when perceived costs of one’s own social distancing behavior increased but trust in other people’s social distancing behavior decreased. Trust in others’ social distancing behavior was additionally negatively related to the motivations for providing one’s own infection status to a contact tracing app and for using the Data Donation app. Hence, when people have the impression that their fellow human beings are being less solidary by adhering less to recommended or even prescribed behaviors, they apparently attempt to compensate for this tendency by donating their personal data without receiving any direct counter value. However, participants’ motivation for use, providing the requested data, and accepting mandatory use were higher for the contact tracing app than for the Data Donation app. Consequently, people are still more motivated to use the more personally useful app. Interestingly, age was negatively related to the motivation for using the Data Donation app. This result may indicate young people’s generally higher willingness to use apps, as already shown by Cho [45]. However, when participants were explicitly asked how willing they were to provide the personal, health, and activity data requested by this app (although this was already highlighted in the app description), this willingness was not correlated with age (*r*=–.02, *P*=.74). Apparently, it is important to be as transparent as possible when indicating which specific data are collected by an app to create the basis for truly informed consent. Finally, the perceived severity of and vulnerability to data misuse were negatively related to the participants’ motivation for using a contact tracing app; meanwhile, only vulnerability to data misuse was negatively related to the motivation for providing one’s own infection status to a contact tracing app and for using the Data Donation app. Also, the participants’ general trust in official app providers was the most important independent variable with respect to app use motivation and data provision and donation. This result emphasizes the significant role of data security issues and trust in the context of app-based measures to combat the current pandemic.

### Limitations

Some limitations of the present study should be mentioned. First, these are cross-sectional, correlational data that do not allow conclusions regarding causal relationships between the independent and dependent variables of the regression models. Second, although the present sets of psychological variables explained substantial interindividual variance in the participants’ motivation for social distancing, using apps, and providing personal data, situational factors may also play important roles. For example, a high motivation for social distancing may be counteracted by insufficient space in urban infrastructure and public transport, while app use motivation also depends on the availability of the required hardware, usability of the software, and reliability of the data processing. Third, the participants’ behavioral motivations were observed rather than their actual behavior. At the time of the study, an official contact tracing app was not yet available but had already been announced by the German Federal Government. The app has since been released (June 16, 2020); therefore, the actual download frequency and behavioral data of the app will be available for future research. Fourth, and relatedly, open questions remain as to whether different providers are assessed as having different levels of trust and how this could influence the relationships observed. Fifth, the present results are based on linear regression analyses, as the statistical assumptions were met, and all rating scales were treated as metric (except gender as a nominal variable), including the dependent variables. However, ordinal regression models may be considered as an alternative approach (with different limitations) which, however, largely replicated the present results for all models (see Multimedia Appendix 1). Finally, the participants in this sample had a mean age of approximately 33 years and were all residents of Germany; therefore, the generalizability of the present results to older people and other countries or cultures should be applied with caution.

### Conclusion

The present study revealed four key findings. First, the present models revealed some important cognitive factors that constitute people’s motivation for social distancing and using apps to combat the COVID-19 pandemic. However, the reduced model assessing the motivation for social distancing explained more interindividual variance than the extended model addressing app use, indicating that app use is more strongly constituted by other factors. Second, in addition to processes of threat and coping appraisal, social trust was found to be a relevant factor, highlighting the importance of both interpersonal solidarity and data security issues in the context of the ongoing pandemic. Third, the focus of the present study was on the joint contribution of several independent variables to the motivations for social distancing, using an app, and providing health-related data. As a consequence, several bivariate correlations between independent and dependent variables disappeared when independent variables were considered simultaneously in the regression models (cf. Multimedia Appendix 1). This result indicates that health campaigns addressing complex cognitive appraisal processes may fall short when focusing on selected correlations between individual variables. Finally, participants preferred the use of a contact tracing app compared to a pure data donation app that has no direct utility for its users.

## Appendix

Multimedia Appendix 1 Bivariate correlations between independent and dependent variables of the regression models, robustness checks, and partial regression plots.

## Abbreviations

COVID-19: coronavirus disease
DFG: Deutsche Forschungsgemeinschaft
OLS: ordinary least squares
PEPP-PT: Pan-European Privacy-Preserving Proximity Tracing
PMT: protection motivation theory
SARS-CoV-2: severe acute respiratory syndrome coronavirus 2
