# Supplementary material for: Motivations for Social Distancing and App Use as Complementary Measures to Combat the COVID-19 Pandemic: Quantitative Survey Study
Source: J Med Internet Res. 2020 Aug 27;22(8):e21613. doi: 10.2196/21613 (PMC7458661; doi:10.2196/21613)
Supplement: Multimedia Appendix 1 [file jmir_v22i8e21613_app1.pdf]

Multimedia Appendix 1

Motivations for Social Distancing and App Use as Complementary Measures to Combat the COVID-19 Pandemic: Quantitative Survey Study

Kai Kaspar

A: Bivariate correlations between independent and dependent variables

Bivariate correlations between independent and dependent variables of the regression models are depicted in Table MA1. Please note that that bivariate correlations should be interpreted with caution in the case of multiple regression analyses. For example, on the level of bivariate correlations, participants' age, perceived vulnerability to an infection, and day of participation showed a positive relation to the motivation for social distancing, whereas perceived response costs of social distancing showed a negative correlation. In the complete model that simultaneously considered all independent variables, however, these significant relations disappeared.

Table MA1: Bivariate Pearson correlations and associated *P* values between independent and dependent variables of the regression models.

Independent Variable	Motivation for social distancing		Motivation for using a contact tracing app		Motivation for providing the infection status to a contact tracing app		Motivation for using the Data Donation app	
	r	*P*	*r*	*P*	*r*	*P*	*r*	*P*
1. Age	.121	.015	-.003	.945	-.024	.628	-.113	.023
2. Gender (0 = male, 1 = female)	.061	.217	-.006	.905	.026	.598	.053	.285
3. Severity of infection	.271	<.001	.152	.002	.107	.032	.073	.144
4. Vulnerability to infection	.376	<.001	.274	<.001	.235	<.001	.181	<.001
5. Rewards of avoiding social distancing	-.518	<.001	-.185	<.001	-.206	<.001	-.167	.001
6. Self-efficacy regarding social distancing	.529	<.001	.209	<.001	.183	<.001	.082	.099
7. Response efficacy of social distancing	.645	<.001	.346	<.001	.326	<.001	.256	<.001
8. Response costs of social distancing	-.207	<.001	.003	.957	-.059	.236	-.050	.316
9. Trust in others people's social distancing behavior	.219	<.001	-.014	.780	-.021	.679	-.059	.234
10. Day of participation	-.131	.008	-.059	.235	-.034	.499	-.026	.604
11. Severity of data misuse			-.285	<.001	-.287	<.001	-.271	<.001
12. Vulnerability to data misuse			-.492	<.001	-.496	<.001	-.454	<.001
13. General trust in official app providers			.580	<.001	.573	<.001	.514	<.001

B: Testing the assumptions of the linear regression models

Statistical assumptions were checked for all regression models to assess the robustness of the results [1]. Across models, no outliers were found by means of Cook's distance (max. distance = 0.13) using a threshold value of 1 [2] and by means of leverage points (max. value = 0.16) using a threshold value of 0.2 [3]. Also, no multicollinearity was present (max. VIF = 1.91), given a threshold value of 10 [4]. The linearity and normality assumptions were visually inspected and generally met, but the Shapiro-Wilk test showed a significant result in two out of the four regression models. However, the present sample size was sufficiently large so that the regression models can be considered robust to violations of the normality assumption [5, 6]. The Durbin-Watson test showed independence among error terms, given a target value of 2 [7]. Although OLS estimates are unbiased in the presence of heteroscedasticity, significance tests may lead to biased results under such circumstances. Scatterplots were visually inspected and indicated noticeable deviation from homoscedasticity only for the regression model assessing participants' motivation for social distancing. Also, the Breusch-Pagan test showed a significant result in this case. Hence, the robust HC3 estimator was used for significance testing in this model [8], while the standard OLS estimator was preferred for the other models due to its superiority given homoscedasticity [9]. In general, the different accounts only slightly differed regarding the observed *P* values (see Table MA2). Additionally, bolded values in Table MA2 indicate significant relations that changed to non-significant relations (.05 < *P* < .10), or vice versa (*P* < .05), when applying an ordinal regression analysis to the data instead of the preferred linear regression analysis. All analyses were computed with SPSS 25.

Table MA2: Comparison of *P* values based on the standard OLS estimates (P_{OLSE}) and the heteroscedasticity-robust HC3 estimator (P_{HC3}), including the *P* values of the Breusch-Pagan screening test for heteroscedasticity.

Independent Variable	Motivation for social distancing		Motivation for using a contact tracing app		Motivation for providing the infection status to a contact tracing app		Motivation for using the Data Donation app	
	P_{OLSE}	P_{HC3}	P_{OLSE}	P_{HC3}	P_{OLSE}	P_{HC3}	P_{OLSE}	P_{HC3}
1. Age	.354	.337	.608	.611	.736	.743	.050	.077
2. Gender (0 = male, 1 = female)	.667	.700	.218	.241	.849	.856	.516	.536
3. Severity of infection	.004	.003	**.089**	.121	.558	.577	.434	.445
4. Vulnerability to infection	.739	.743	.138	.160	.402	.433	.770	.762
5. Rewards of avoiding social distancing	<.001	<.001	.698	.718	.263	.280	.228	.313
6. Self-efficacy regarding social distancing	<.001	<.001	.006	.002	.061	.091	.882	.891
7. Response efficacy of social distancing	<.001	<.001	.045	.032	**.066**	.097	.105	.085
8. Response costs of social distancing	.436	.406	.001	.002	.180	.221	.374	.446
9. Trust in others people's social distancing behavior	.001	.003	**.046**	.072	**.032**	.059	.017	.023
10. Day of participation	.472	.532	.286	.311	.767	.780	.530	.507
11. Severity of data misuse			.031	.052	**.109**	.125	.161	.215
12. Vulnerability to data misuse			<.001	<.001	<.001	<.001	.001	.002
13. General trust in official app providers			<.001	<.001	<.001	<.001	<.001	<.001
P-value of the Breusch-Pagan test		<.001		.862		.053		.195

C1: Partial regression plots of model “Motivation for social distancing”

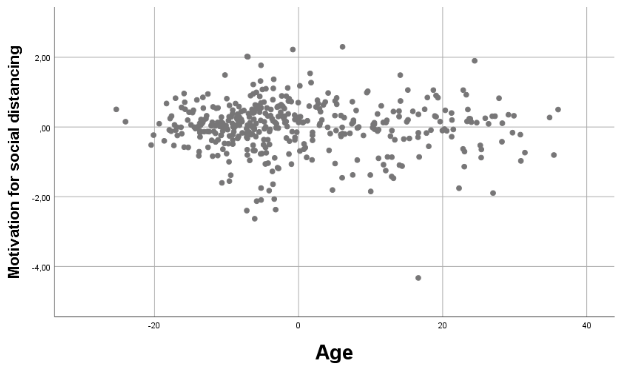

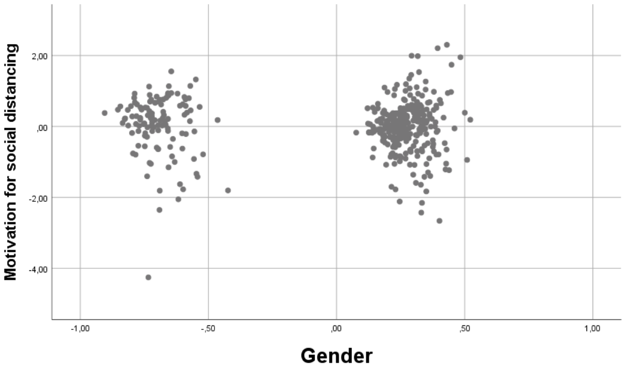

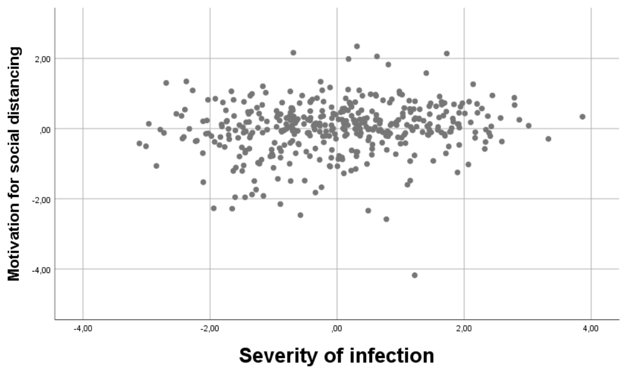

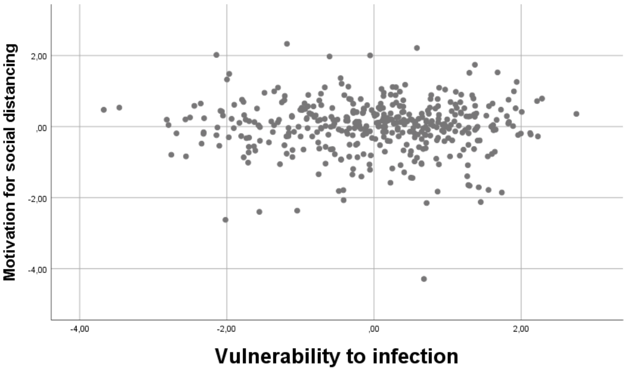

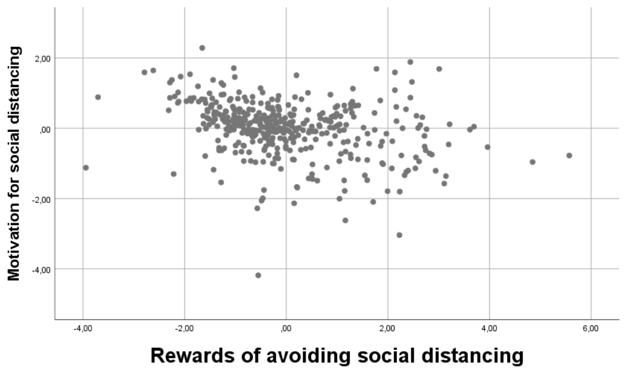

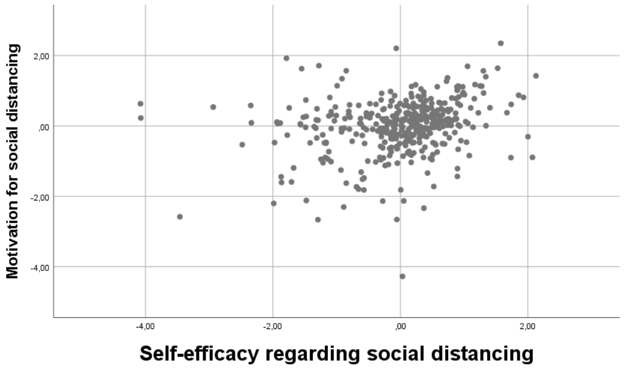

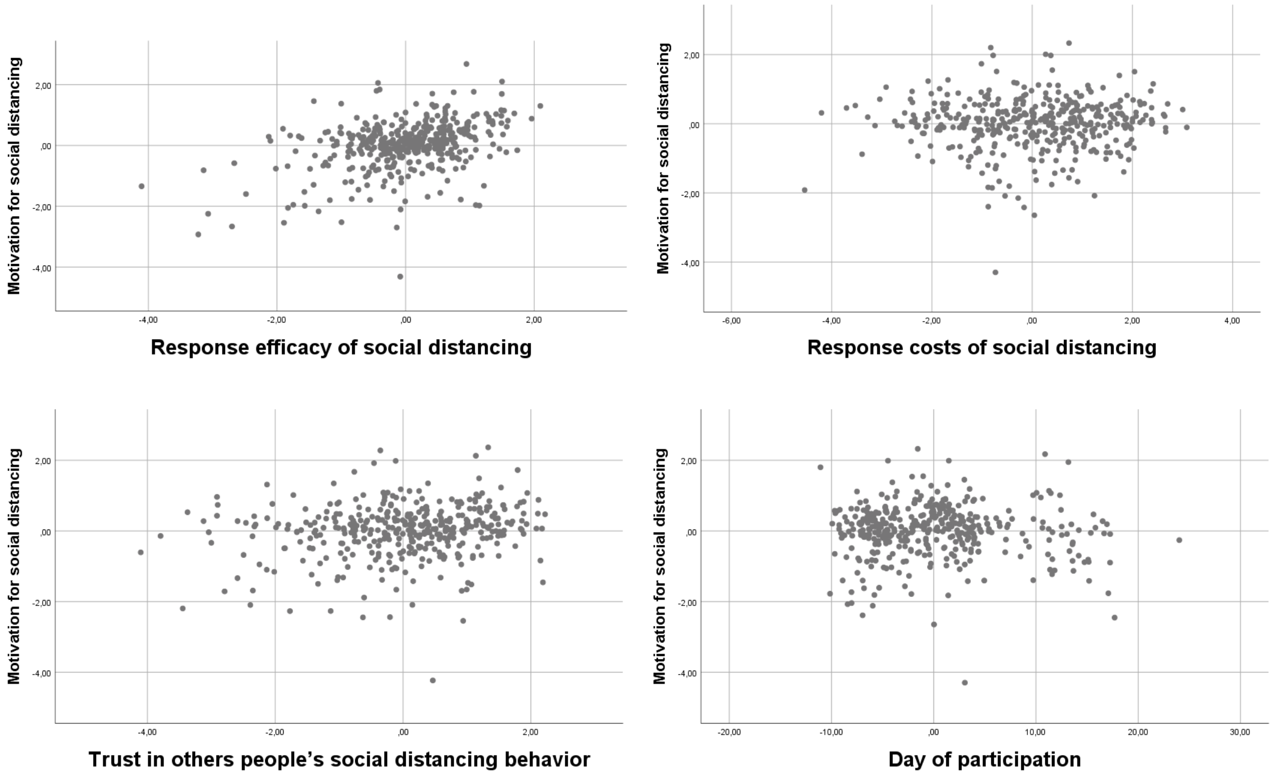

C2: Partial regression plots of model “Motivation for using a contact tracing app”

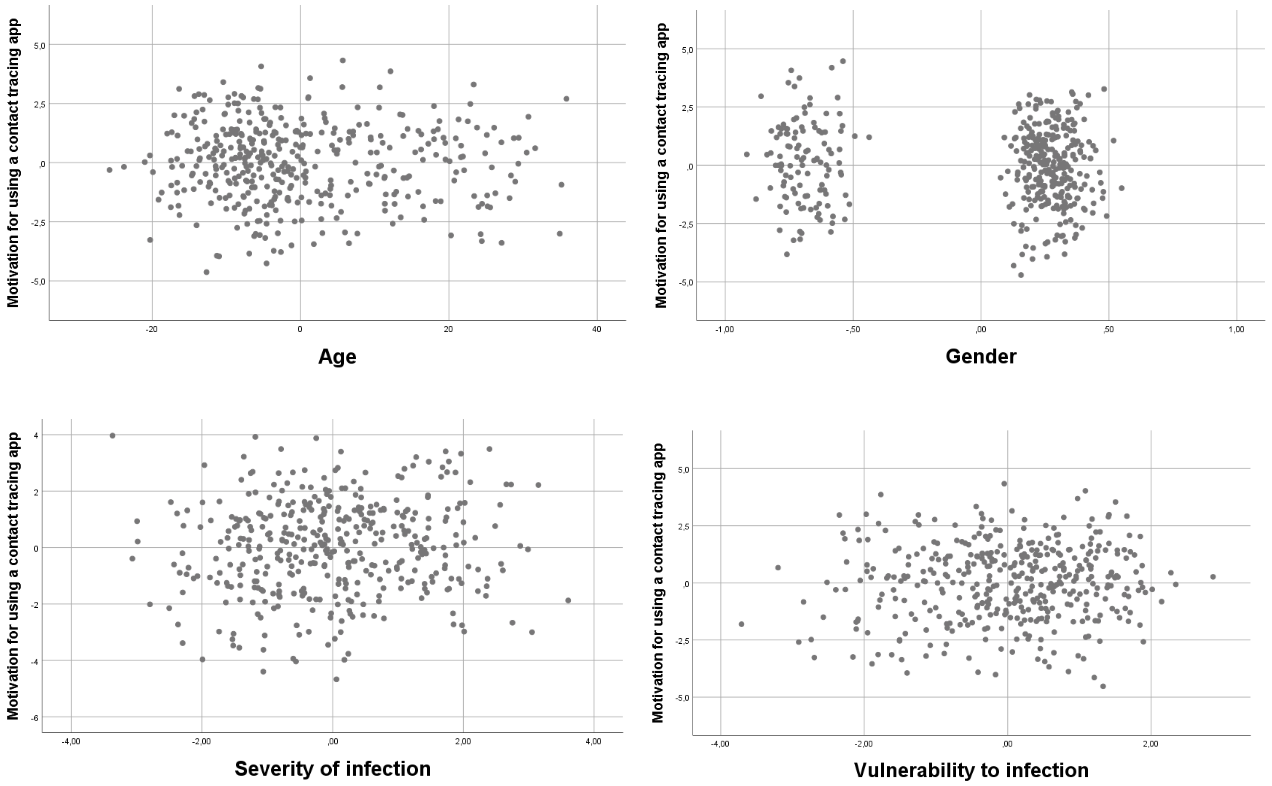

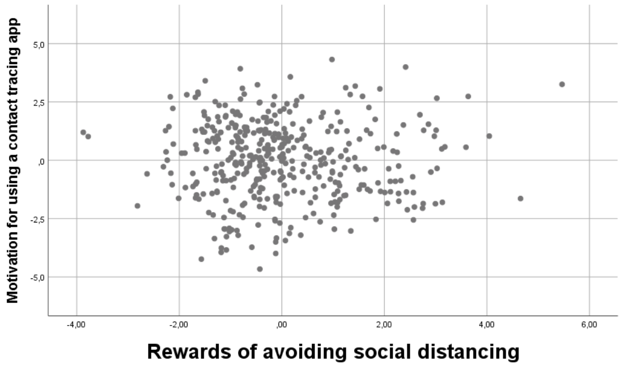
Motivation for using a contact tracing app
Rewards of avoiding social distancing

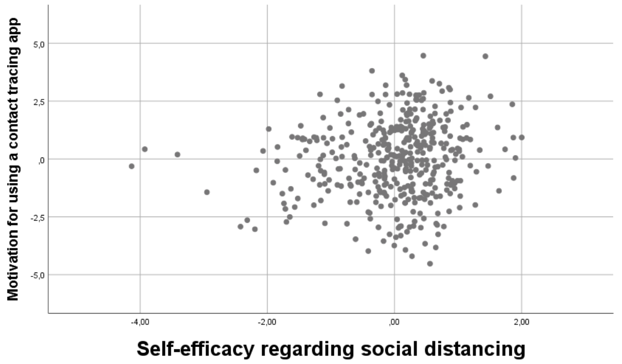
Motivation for using a contact tracing app
Self-efficacy regarding social distancing

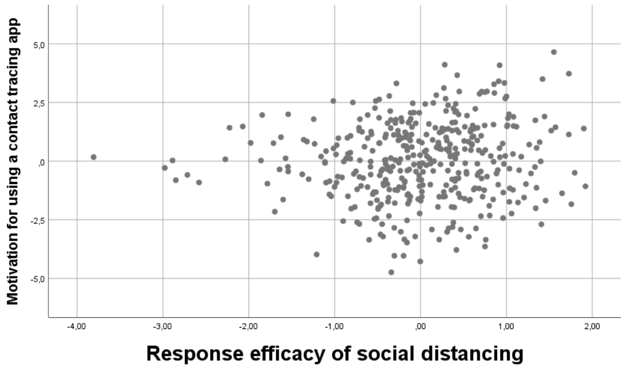
Motivation for using a contact tracing app
Response efficacy of social distancing

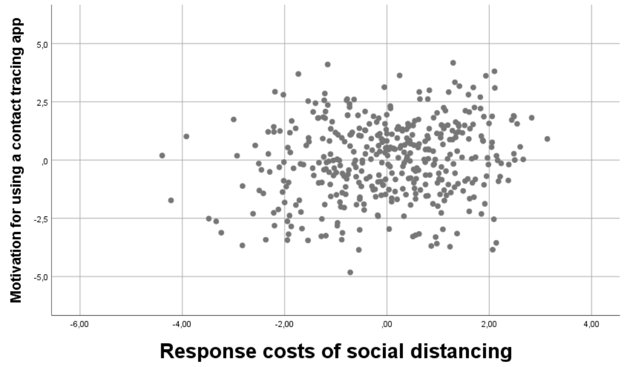
Motivation for using a contact tracing app
Response costs of social distancing

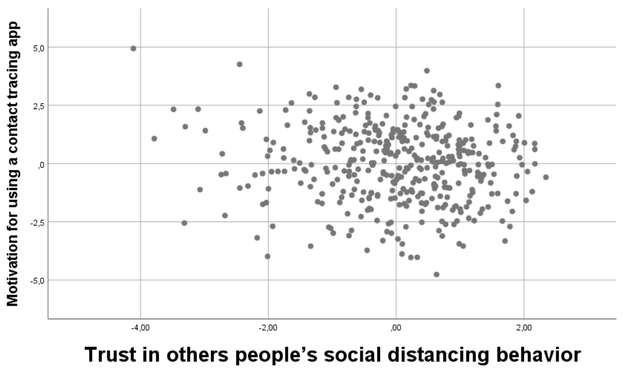
Motivation for using a contact tracing app
Trust in others people’s social distancing behavior

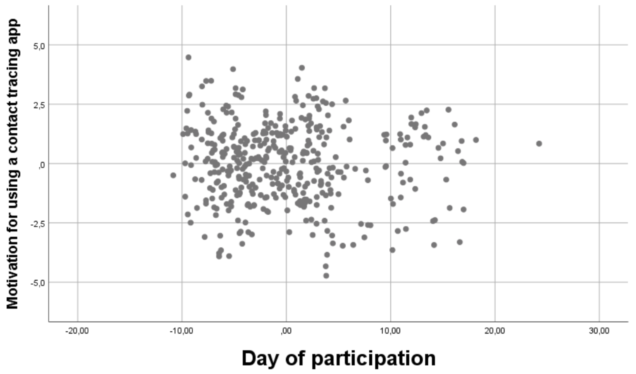
Motivation for using a contact tracing app
Day of participation

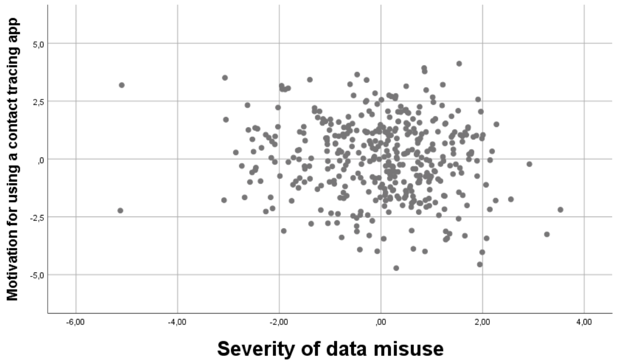
Motivation for using a contact tracing app
Severity of data misuse

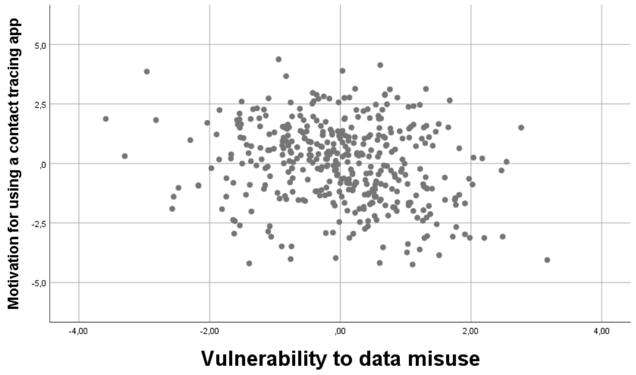
Motivation for using a contact tracing app
Vulnerability to data misuse

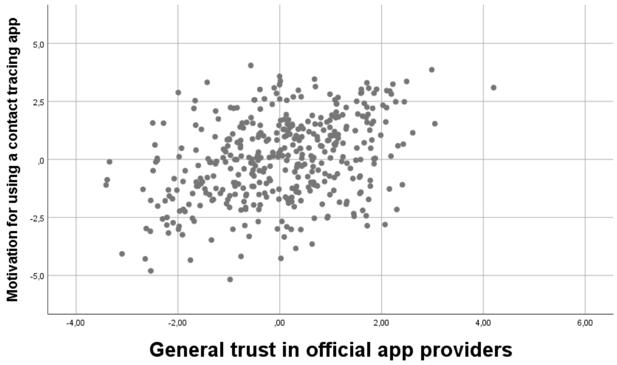

C3: Partial regression plots of model "Motivation for providing the infection status to a contact tracing app"

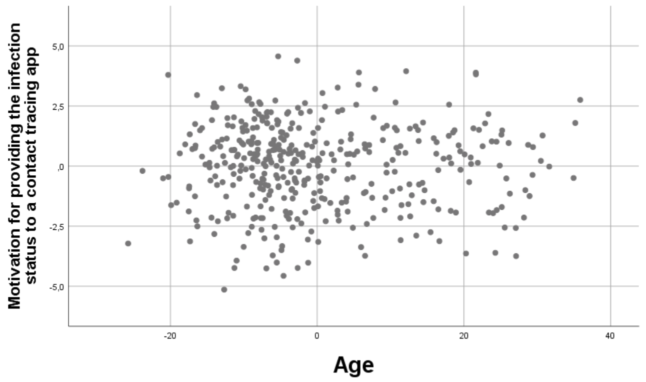

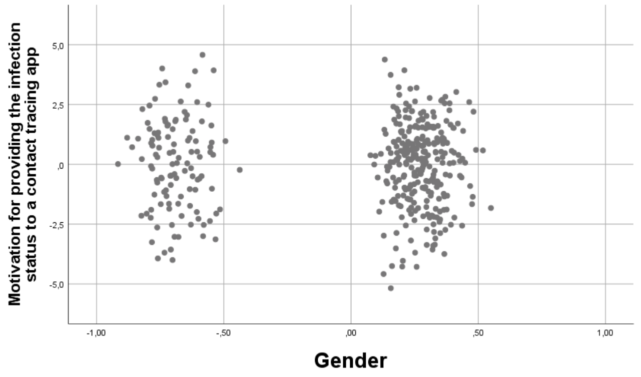

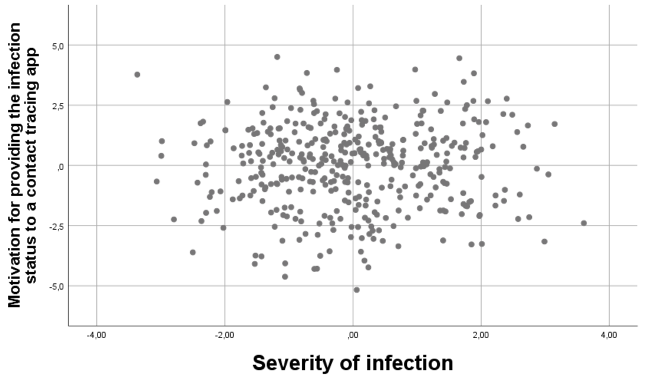

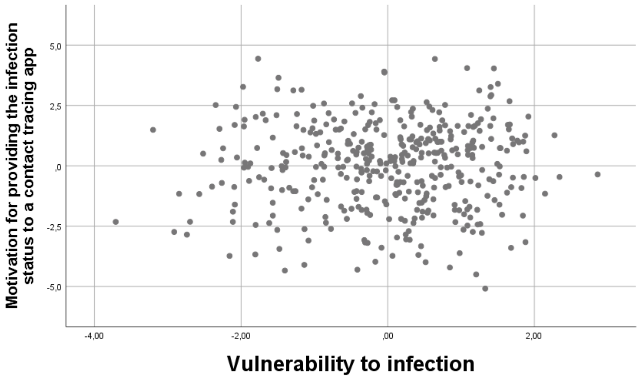

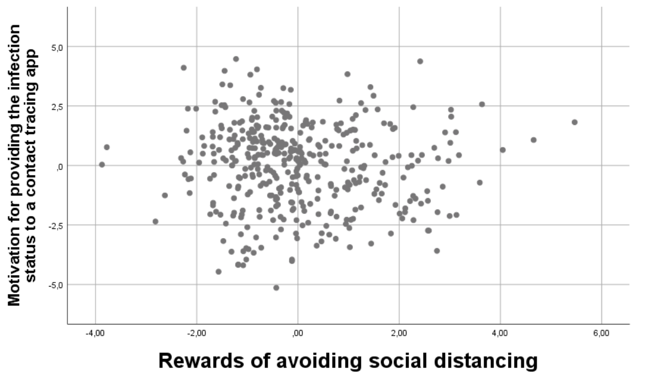

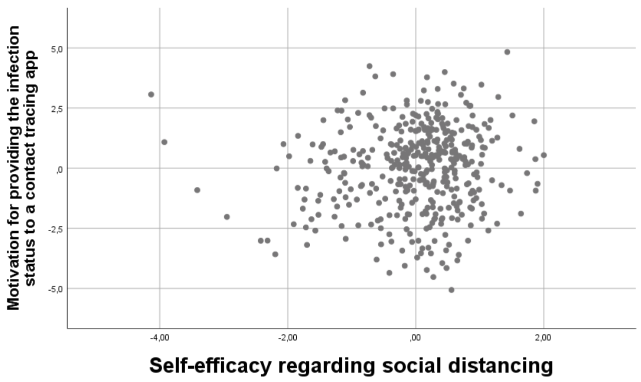

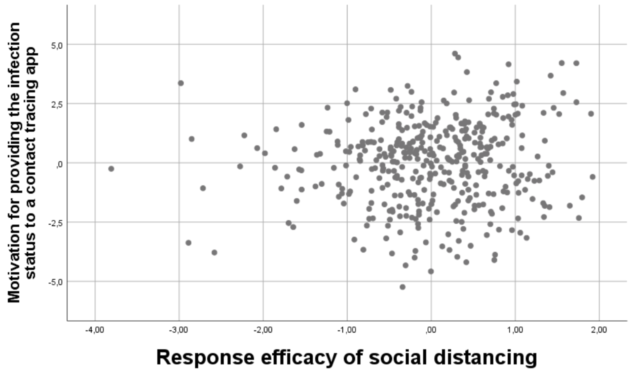
Motivation for providing the infection status to a contact tracing app
5,0
2,5
,0
-2,5
-5,0
-4,00
-3,00
-2,00
-1,00
,00
1,00
2,00
Response efficacy of social distancing

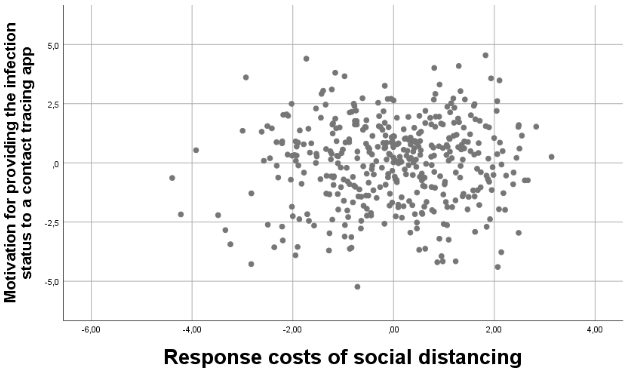
Motivation for providing the infection status to a contact tracing app
5,0
2,5
,0
-2,5
-5,0
-6,00
-4,00
-2,00
,00
2,00
4,00
Response costs of social distancing

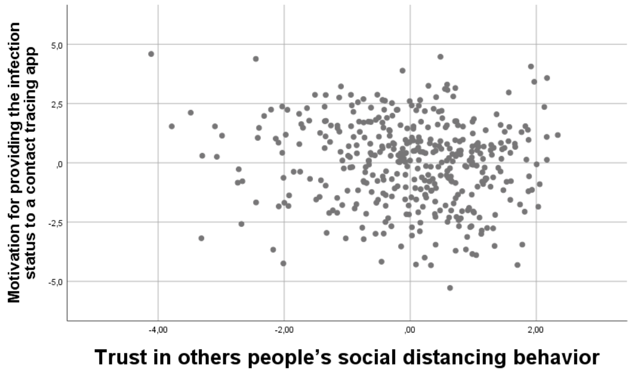
Motivation for providing the infection status to a contact tracing app
5,0
2,5
,0
-2,5
-5,0
-4,00
-2,00
,00
2,00
Trust in others people's social distancing behavior

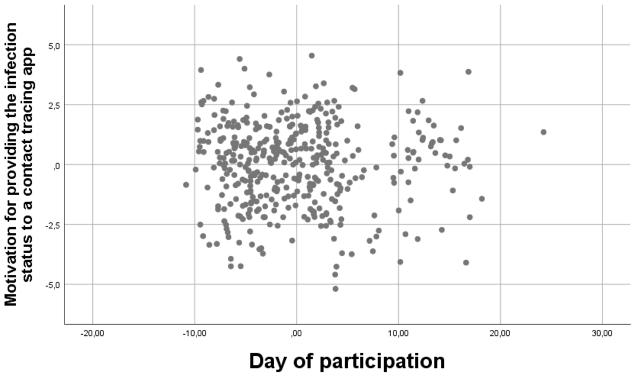
Motivation for providing the infection status to a contact tracing app
5,0
2,5
,0
-2,5
-5,0
-20,00
-10,00
,00
10,00
20,00
30,00
Day of participation

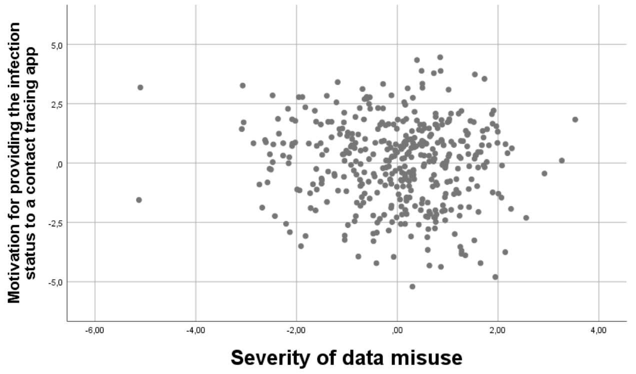
Motivation for providing the infection status to a contact tracing app
5,0
2,5
,0
-2,5
-5,0
-6,00
-4,00
-2,00
,00
2,00
4,00
Severity of data misuse

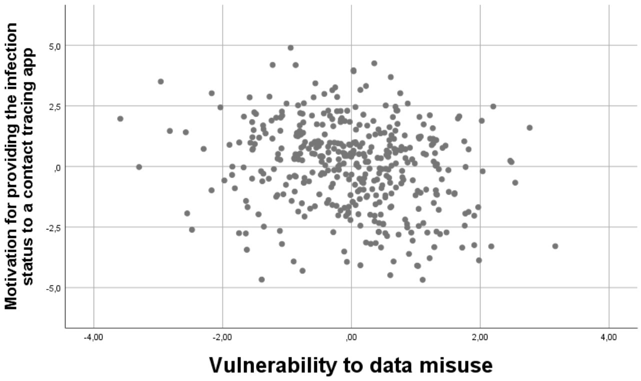
Motivation for providing the infection status to a contact tracing app
5,0
2,5
,0
-2,5
-5,0
-4,00
-2,00
,00
2,00
4,00
Vulnerability to data misuse

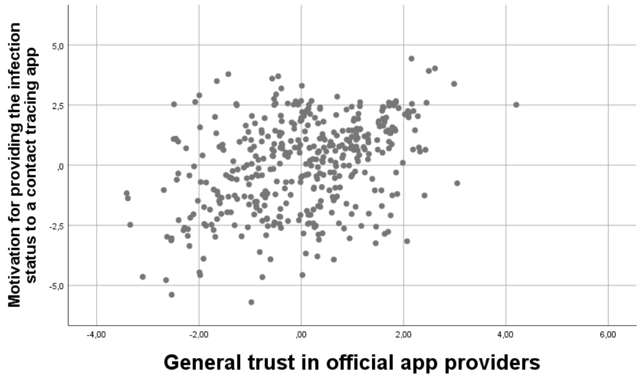
Motivation for providing the infection status to a contact tracing app
5,0
2,5
,0
-2,5
-5,0
-4,00
-2,00
,00
2,00
4,00
6,00
General trust in official app providers

C4: Partial regression plots of model "Motivation for using the data donation app"

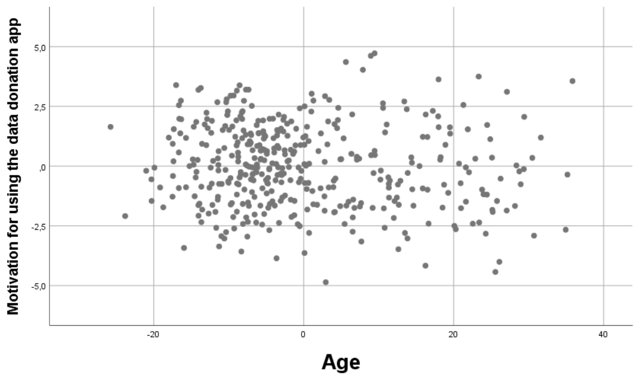

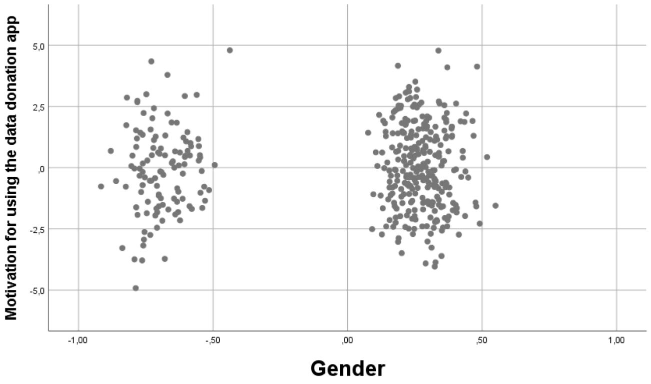

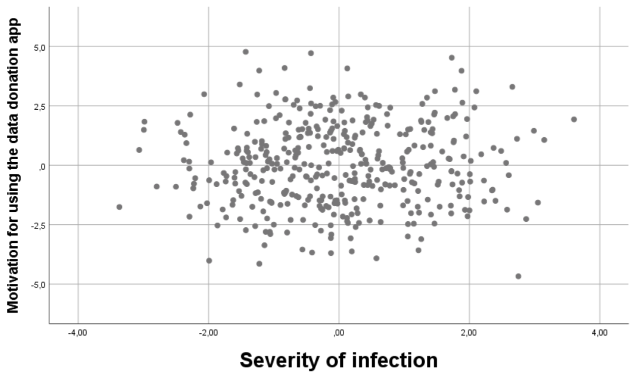

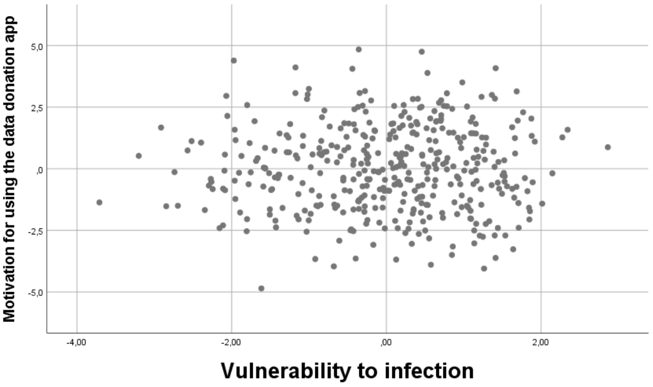

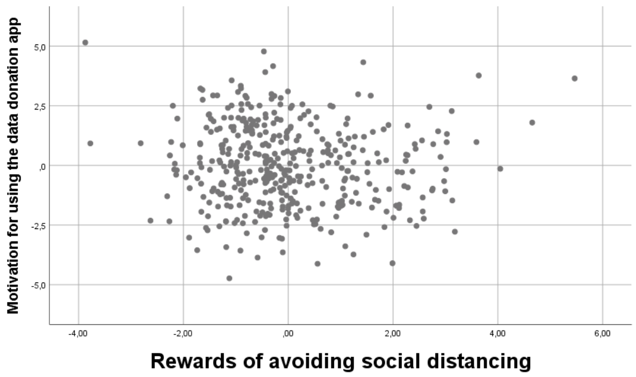

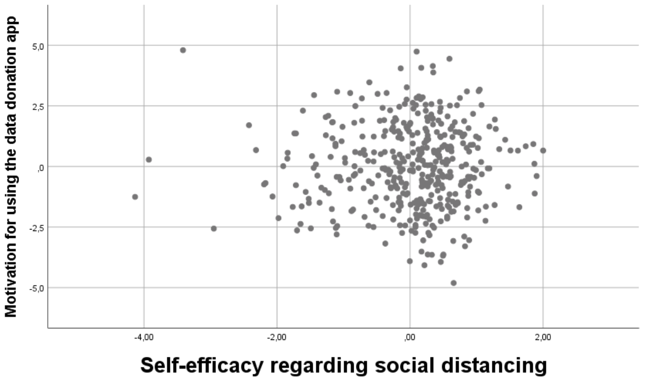

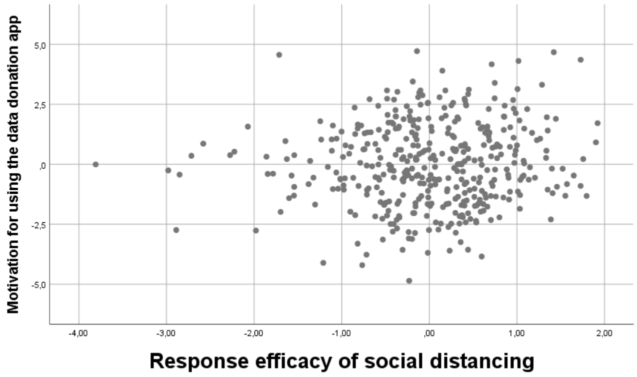

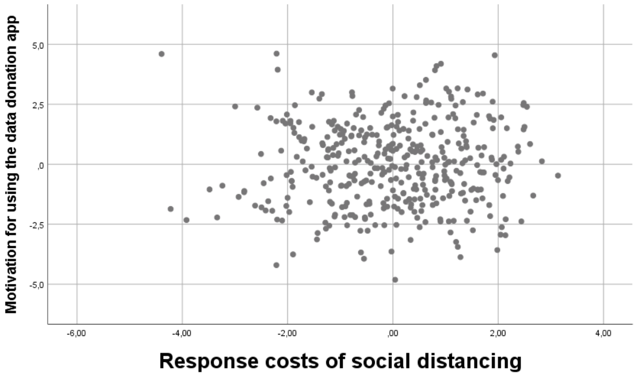

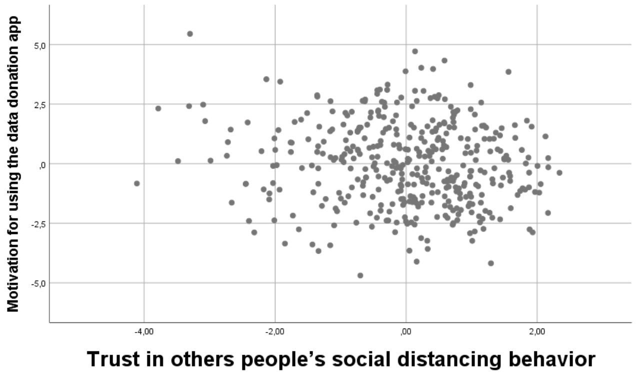
Motivation for using the data donation app
5,0
2,5
,0
-2,5
-5,0
-4,00
-2,00
,00
2,00
Trust in others people's social distancing behavior

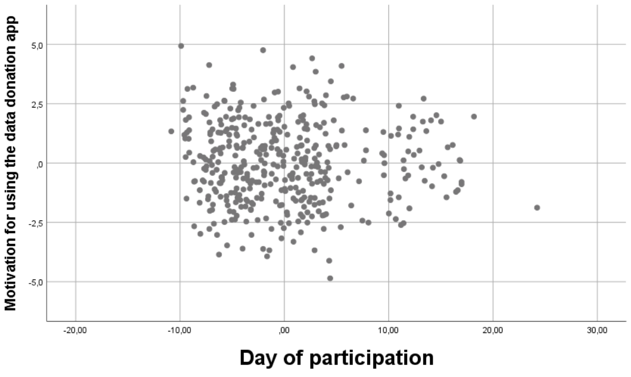
Motivation for using the data donation app
5,0
2,5
,0
-2,5
-5,0
-20,00
-10,00
,00
10,00
20,00
30,00
Day of participation

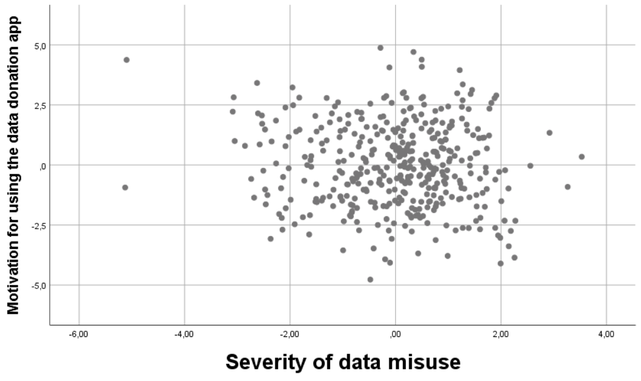
Motivation for using the data donation app
5,0
2,5
,0
-2,5
-5,0
-6,00
-4,00
-2,00
,00
2,00
4,00
Severity of data misuse

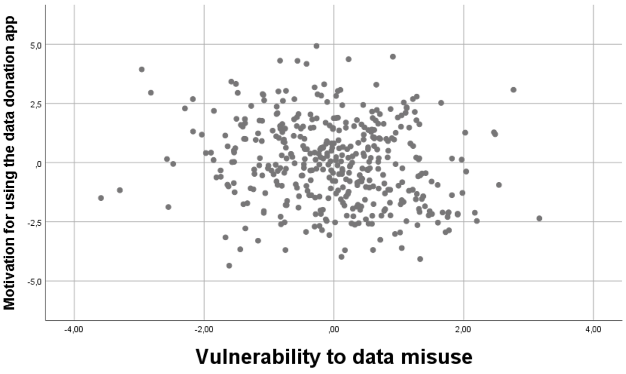
Motivation for using the data donation app
5,0
2,5
,0
-2,5
-5,0
-4,00
-2,00
,00
2,00
4,00
Vulnerability to data misuse

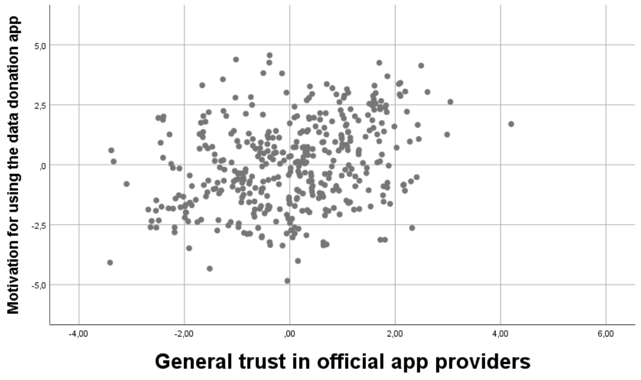
Motivation for using the data donation app
5,0
2,5
,0
-2,5
-5,0
-4,00
-2,00
,00
2,00
4,00
6,00
General trust in official app providers

References

[1] Poole MA, O'Farrell PN. The assumptions of the linear regression model. Trans Inst Br Geogr 1971 Mar; 52:145-158. https://doi.org/10.2307/621706

[2] Cook RD, Weisberg S. Residuals and Influence in Regression. New York/London: Chapman & Hall; 1982.

[3] Huber PJ. Robust Statistics. New York: John Wiley; 1981.

[4] Alin A. Multicollinearity. WIREs Comp Stat 2010 May; 2(3):370-374. https://doi.org/10.1002/wics.84

[5] Schmidt AF, Finan C. Linear regression and the normality assumption. J Clin Epidemiol 2018 Jun; 98:146-151. https://doi.org/10.1016/j.jclinepi.2017.12.006

[6] Lumley T, Diehr P, Emerson S, Chen L. The importance of the normality assumption in large public health data sets. Annu Rev Public Health 2002 May; 23(1):151-169. https://doi.org/10.1146/annurev.publhealth.23.100901.140546

[7] Nerlove M, Wallis KF. Use of the Durbin-Watson statistic in inappropriate situations. Econometrica 1966 Jan; 34(1):235-238. http://doi.org/10.2307/1909870

[8] Hayes AF, Cai L. Using heteroskedasticity-consistent standard error estimators in OLS regression: An introduction and software implementation. Behav Res Methods 2007 Nov; 39(4):709-722. https://doi.org/10.3758/BF03192961

[9] Long JS, Ervin LH. Using heteroscedasticity consistent standard errors in the linear regression model. Am Stat 2000 Aug; 54(3):217-224. https://doi.org/10.1080/00031305.2000.10474549
